# Supplementary material for: CRISPR-Cas9 precision editing of kinetochore protein phosphosite codons in Leishmania mexicana
Source: Front Cell Infect Microbiol. 2026 Apr 14;16:1788564. doi: 10.3389/fcimb.2026.1788564 (PMC13121160; doi:10.3389/fcimb.2026.1788564)
Supplement: Supplementary Figure 1 — Additional target mutations generated with the precision editing methodology with their genotypes as detected by PCR and Sanger sequencing. The number of clones per slice is indicated around the outside of each pie, with the total clones assessed below. Yellow halos around PCR screening pie charts indicate clones selected for sequencing. The combined data for all mutations generated for both PCR screening and Sanger sequencing is shown at the bottom. For PCR data: WT – PCR product was detected in the WT primer set reaction and not in the mutant set reaction; Heterozygous – PCR product was detected in both WT and mutant primer set reactions with approximately equivalent intensity; Homozygous – PCR product was only detected in mutant primer set reaction; Unclear – PCR product was detected in both WT and mutant primer sets with either differing intensity in each or additional unknown products; Fail – no PCR product was detected in either reaction. For Sanger sequencing data: WT – both alleles match the reference sequence; Heterozygous – one allele matched the reference sequence, one allele matched the repair template sequence (identified by dual peaks of similar height in the chromatogram); Homozygous – both alleles match the repair template sequence; Complex – evidence of integration of the repair template either to different extents on each allele, or with unexpected mutations; Fail – the sequence was unable to align with either the reference sequence or the repair template sequence. [file DataSheet1.docx]

CRISPR-Cas9 precision editing of kinetochore protein phosphosite codons in *Leishmania mexicana*

Charlotte McNiven^1^, Juliana B. T. Carnielli^1^, Vincent Geoghegan^1^, Joana R.C. Faria^1^, Jeremy C. Mottram^1*^

^1^ York Biomedical Research Institute and Department of Biology, University of York, UK.

* for correspondence

Contents

[Supplementary Figure 1 2](#_Toc225238079)

[Supplementary Table 1 4](#_Toc225238080)

[Supplementary Table 2 5](#_Toc225238081)

[Supplementary Table 3 12](#_Toc225238082)

[Supplementary Table 4 13](#_Toc225238083)

# Supplementary Figure 1


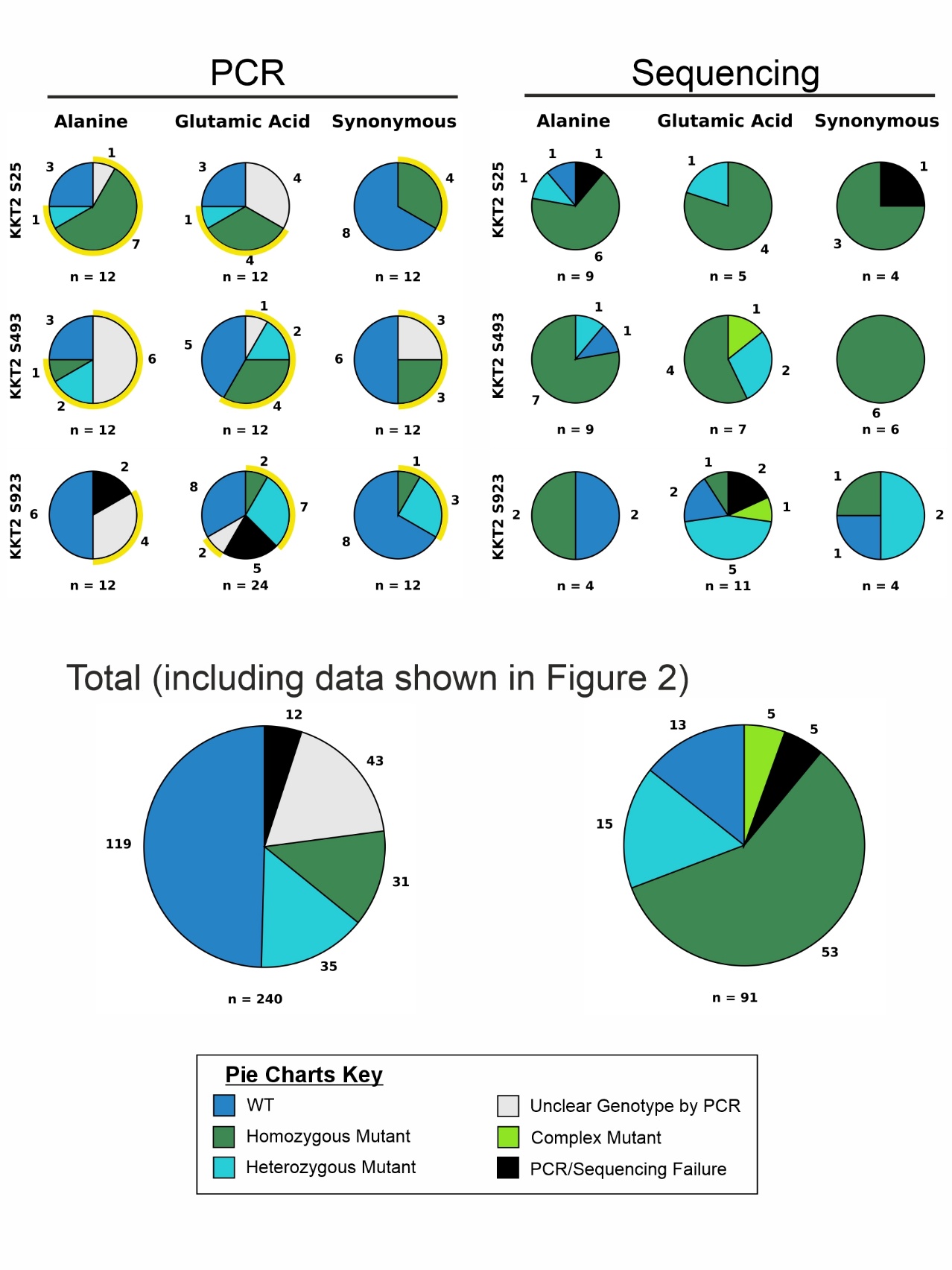


**Supplementary Figure 1.** Additional target mutations generated with the precision editing methodology with their genotypes as detected by PCR and Sanger sequencing. The number of clones per slice is indicated around the outside of each pie, with the total clones assessed below. Yellow halos around PCR screening pie charts indicate clones selected for sequencing. The combined data for all mutations generated for both PCR screening and Sanger sequencing is shown at the bottom. For PCR data: WT – PCR product was detected in the WT primer set reaction and not in the mutant set reaction; Heterozygous – PCR product was detected in both WT and mutant primer set reactions with approximately equivalent intensity; Homozygous – PCR product was only detected in mutant primer set reaction; Unclear – PCR product was detected in both WT and mutant primer sets with either differing intensity in each or additional unknown products; Fail – no PCR product was detected in either reaction. For Sanger sequencing data: WT – both alleles match the reference sequence; Heterozygous – one allele matched the reference sequence, one allele matched the repair template sequence (identified by dual peaks of similar height in the chromatogram); Homozygous – both alleles match the repair template sequence; Complex – evidence of integration of the repair template either to different extents on each allele, or with unexpected mutations; Fail – the sequence was unable to align with either the reference sequence or the repair template sequence.

# Supplementary Table 1

Kinetochore phosphosite data from Geoghegan et al. (2022) used to determine target sites of interest.

| Target | Change during cell cycle | Reduced by AB1? | Notes |
| --- | --- | --- | --- |
| KKT2 S25 | No change | Yes | Not proximal to KKT3 |
| KKT2 S493 | Decreases | No |  |
| KKT2 S530 | Increases | Yes |  |
| KKT2 S923 | No change | Yes | Confident localisation |
| KKT4 S422 | Increases | Yes | In microtubule binding region, confident localisation |
| KKT7 S304 | Increases | Yes |  |

# Supplementary Table 2

Oligonucleotide sequences used in this study. All sequences are provided in the 5’ to 3’ orientation.

|  | **Primer Name** | **Target** | **Primer Purpose** | **Sequence** |
| --- | --- | --- | --- | --- |
| Single-stranded Repair | OL12999 | KKT2 S493A | ssODN repair template | GTCTCAGAGGTTGCAGATCGCGAGGAAGCCGCGCCACGGACCTCACGTTCTGTCCGTCGTAGCGTAGCGCTAACCGAACAAGAACGTGGCAGACTTGTGCGTTCTAGCCCGGTCCAGTAC |
|  | OL12993 | KKT2 S493A | Forward Screening Primer – PCR construct for restriction digest | TACGGTGCTGGTAGGGATGA |
|  | OL12994 | KKT2 S493A | Reverse Screening Primer – PCR construct for restriction digest | TGTCATTACGTGCCCGTTCA |
|  | OL12932 | KKT2 S923A | ssODN repair template | AAGCAAGCCATCATGCCGCCTCAAGTGCCACGCGGACGAGCACAGCAGCCACGTGCGCCAGCGGTTTCGGGTCATACGGCTCAAGGTGGTCCGCCACTGCCGCGCCGCGGCCCAGCTGCG |
|  | OL12934 | KKT4 S422A | ssODN repair template | CTGCAGGGCAGCGCCGATCGTGTCGTCCAGGGGCGTCGTGGCGTTGCGGCGACCAAGGCGGAGACGGCGCCGGCCTATATTACGACGCCCACGCCCGCCGGCAAGGCGTCCACCGCGCTC |
|  | OL12901 | KKT7 S304 | sgRNA (post-transfection this guide was identified as mistakenly targeting the repair sequence rather than the WT) | gaaattaatacgactcactataggATATCGACCGACGACGCTGAgttttagagctagaaatagc |
|  | OL12936 | KKT7 S304A | ssODN repair template | GCGAAGCAACAGAGTCGCGTTCACTCTTCTGCGCACCAACGGCGGCGTAGCATTAGCATTGTCGCGGCGGATGCGCTTGCCAAATCGGGCGAGGACGAAGACGGTGACGACAACGACACC |
|  | OL12937 | KKT7 S304S | ssODN repair template | GCGAAGCAACAGAGTCGCGTTCACTCTTCTGCGCACCAACGGCGGCGTAGCATTAGCATTGTCTCGGCGGATGCGCTTGCCAAATCGGGCGAGGACGAAGACGGTGACGACAACGACACC |
| Double-stranded Repair | OL12632 | KKT2 S25 | sgRNA | gaaattaatacgactcactataggGGGGTCTGCGACAAATCACGgttttagagctagaaatagc |
|  | OL14011 | KKT2 S25 | sgRNA | gaaattaatacgactcactataggACCCCCGCCATCTCTCGACTgttttagagctagaaatagc |
|  | OL14224 | KKT2 S25A | Double-stranded DNA Repair Template Primer 1 | GGCCTCTGATGTCACACTTTTGCGGCTCGTTGTCGAGGACTCCACCACGGGGTGGGGCGATATCTATGCCCAGAGACCTTGCGCAAACACCAGCGATTTCACG |
|  | OL14226 | KKT2 S25E | Double-stranded DNA Repair Template Primer 1 | GGCCTCTGATGTCACACTTTTGCGGCTCGTTGTCGAGGACTCCACCACGGGGTGGGGCGATATCTATGCCCAGAGACCTTGAGCAAACACCAGCGATTTCACG |
|  | OL14225 | KKT2 S25A and KKT2 S25E | Shared Double-stranded DNA Repair Template Primer 2 | TCTGCTTGGTCAACAACACATTTTTGGATGTGCGGCGTCTTCACCGTGCTCCCCAGGCGTGAAATCGCTGGTGTTTG |
|  | OL14145 | KKT2 S25S | Double-stranded DNA Repair Template Primer 1 | GGCCTCTGATGTCACACTTTTGCGGCTCGTTGTCGAGGACTCCACCACGGGGTGGGGCGATATCTATGCCCAGAGACCTTAGTCAAACACC |
|  | OL14146 | KKT2 S25S | Double-stranded DNA Repair Template Primer 2 | TCTGCTTGGTCAACAACACATTTTTGGATGTGCGGCGTCTTCACCGTGCTCCCCAGGCGTGAAATCGCTGGTGTTTGACTAAGGTCTCT |
|  | OL14209 | KKT2 S25 | WT Screening Primer 1 | CTATGCCGCGTGATTTGTCG |
|  | OL13859 | KKT2 S25A | Mutant Screening Primer 1 | CAGAGACCTTGCGCAAACAC |
|  | OL14242 | KKT2 S25E | Mutant Screening Primer 1 | CTATGCCCAGAGACCTTGAG |
|  | OL14205 | KKT2 S25S | Mutant Screening Primer 1 | GACCTTAGTCAAACACCAGCG |
|  | OL13860 | KKT2 S25 | Shared Screening Primer 2 and Sequencing Primer | CAGCCCAGTGGTAACTACTC |
|  |  |  |  |  |
|  | OL14228 | KKT2 S493A | Double-stranded DNA Repair Template Primer 1 | CGCCCCGGGTACACCACTGCGTACTGGACCGGGCTAGAACGCACAAGTCTGCCACGTTCTTGTTCGGTTAGCGCTACGCTACGACGGACAGAACGTG |
|  | OL14229 | KKT2 S493E | Double-stranded DNA Repair Template Primer 1 | CGCCCCGGGTACACCACTGCGTACTGGACCGGGCTAGAACGCACAAGTCTGCCACGTTCTTGTTCGGTTAGCTCTACGCTACGACGGACAGAACGTG |
|  | OL14227 | KKT2 S493A and S493E | Shared Double-stranded DNA Repair Template Primer 2 | GGTGTGGCAGTGTCTCACTGGTCTCAGAGGTTGCAGATCGCGAGGAAGCCGCGCCACGGACCTCACGTTCTGTCCGTCGTAGC |
|  | OL14147 | KKT2 S493S | Double-stranded DNA Repair Template Primer 1 | GGTGTGGCAGTGTCTCACTGGTCTCAGAGGTTGCAGATCGCGAGGAAGCCGCGCCACGGACCTCACGTTCTGTCCGTCGTAGCGTATCTCTAAC |
|  | OL14148 | KKT2 S493S | Double-stranded DNA Repair Template Primer 2 | CGCCCCGGGTACACCACTGCGTACTGGACCGGGCTAGAACGCACAAGTCTGCCACGTTCTTGTTCGGTTAGAGATACGCTACGACG |
|  | OL13964 | KKT2 S493 | WT Screening Primer 1 | CTCCGTTAAGCTGACGCTAC |
|  | OL13862 | KKT2 S493A | Mutant Screening Primer 1 | CTTGTTCGGTTAGCGCTACG |
|  | OL14243 | KKT2 S493E | Mutant Screening Primer 1 | CGTTCTTGTTCGGTTAGCTC |
|  | OL14206 | KKT2 S493S | Mutant Screening Primer 1 | GACAGAACGTGAGGTCCGTG |
|  | OL13861 | KKT2 S493 | Shared Screening Primer 2 | GCTCCAGAAGCTCAACTTGC |
|  | OL12904 | KKT2 S493 | Sequencing Primer 1 | GACTTGTGGAGCGCATGTTG |
|  |  |  |  |  |
|  | OL12902 | KKT2 S530 | sgRNA | gaaattaatacgactcactataggATCTCGTCCGTCATGTCGCGgttttagagctagaaatagc |
|  | OL12903 | KKT2 S530 | sgRNA | gaaattaatacgactcactataggGCAGCGATACTACGGCGCGAgttttagagctagaaatagc |
|  | OL14230 | KKT2 S530A | Double-stranded DNA Repair Template Primer 1 | CGTTCTAGCCCGGTCCAGTACGCAGTGGTGTACCCGGGGCGCGACACTGCGACACGGTGGAATTTGCGGGCGGTTGTAGCGCTCCCTCGGGATATGACCGATG |
|  | OL14232 | KKT2 S530E | Double-stranded DNA Repair Template Primer 1 | CGTTCTAGCCCGGTCCAGTACGCAGTGGTGTACCCGGGGCGCGACACTGCGACACGGTGGAATTTGCGGGCGGTTGTAGAGCTCCCTCGGGATATGACCGATG |
|  | OL14231 | KKT2 S530A and S530E | Shared Double-stranded DNA Repair Template Primer 2 | TCGAGGTCAACTTTGTCATTACGTGCCCGTTCATGCACTTGAACTCGCGTTCAATTTCATCGGTCATATCCCGAGGG |
|  | OL14149 | KKT2 S530S | Double-stranded DNA Repair Template Primer 1 | CGTTCTAGCCCGGTCCAGTACGCAGTGGTGTACCCGGGGCGCGACACTGCGACACGGTGGAATTTGCGGGCGGTTGTAAGTCTCCCTCGGGATATG |
|  | OL14150 | KKT2 S530S | Double-stranded DNA Repair Template Primer 2 | TCGAGGTCAACTTTGTCATTACGTGCCCGTTCATGCACTTGAACTCGCGTTCAATTTCATCGGTCATATCCCGAGGGAGACTTAC |
|  | OL13975 | KKT2 S530 | WT Screening Primer 1 | CCTTCGCGCCGTAGTATCGC |
|  | OL13976 | KKT2 S530A | Mutant Screening Primer 1 | GCGCTCCCTCGGGATATG |
|  | OL14286 | KKT2 S530E | Mutant Screening Primer 1 | ATTTGCGGGCGGTTGTAGAG |
|  | OL14207 | KKT2 S530S | Mutant Screening Primer 1 | ATTTGCGGGCGGTTGTAAGT |
|  | OL13863 | KKT2 S530A and S530S | Mutant Screening Primer 1 | GACCGATGAAATTGAACGCG |
|  | OL13864 | KKT2 S530 | Shared Screening Primer 2 | GCTTTGCGGTTGAGGTGAAG |
|  | OL12905 | KKT2 S530 | Sequencing Primer 1 | CACACATTGCAGTCGAAGCC |
|  |  |  |  |  |
|  | OL14233 | KKT2 S923A | Double-stranded DNA Repair Template Primer 1 | CTGTGGAGGAGCACGTGGTGAAGCAAGCCATCATGCCGCCTCAAGTGCCACGCGGACGAGCACAGCAGCCACGTGCGCCAGCGGTTTCGGGTCATACGGCTCAAG |
|  | OL14235 | KKT2 S923E | Double-stranded DNA Repair Template Primer 1 | CTGTGGAGGAGCACGTGGTGAAGCAAGCCATCATGCCGCCTCAAGTGCCACGCGGACGAGCACAGCAGCCACGTGCGCCAGAGGTTTCGGGTCATACGGCTCAAG |
|  | OL14234 | KKT2 S923A and S923E | Shared Double-stranded DNA Repair Template Primer 2 | AAAGCGGCTGCAGGAGATGGCGCAGCTGGGCCGCGGCGCGGCAGTGGCGGACCACCTTGAGCCGTATGACCCGAA |
|  | OL14151 | KKT2 S923S | Double-stranded DNA Repair Template Primer 1 | CTGTGGAGGAGCACGTGGTGAAGCAAGCCATCATGCCGCCTCAAGTGCCACGCGGACGAGCACAGCAGCCACGTGCGCCAAGTGTTTCGGGTCATAC |
|  | OL14152 | KKT2 S923S | Double-stranded DNA Repair Template Primer 2 | AAAGCGGCTGCAGGAGATGGCGCAGCTGGGCCGCGGCGCGGCAGTGGCGGACCACCTTGAGCCGTATGACCCGAAACACTTGG |
|  | OL14210 | KKT2 S923 | WT Screening Primer 1 | GTGCCCGCTGACGGAG |
|  | OL13865 | KKT2 S923A, S923E and S923S | Mutant Screening Primer 1 | CACCTTGAGCCGTATGACCC |
|  | OL14208 | KKT2 S923S | Mutant Screening Primer 1 | GCCGTATGACCCGAAACACT |
|  | OL7631 | KKT2 S923 | Shared Screening Primer 2 | CTGACTTTCCCAAGGTGAGC |
|  |  |  |  |  |
|  | OL12128 | KKT2 S25 | Sequencing Primer 2 | CTTCCGCTCACTTCCCATGT |
|  |  | KKT2 all sites | Sequencing PCR Forward |  |
|  | OL12616 | KKT2 all sites | Sequencing PCR Reverse | GGTGTCCCCGTGACATCATT |
|  |  |  |  |  |
|  | OL14592 | KKT4 S422A | Double-stranded DNA Repair Template Primer 1 | GCACCACGTTGGACACGTCTCGTCTGCAGGGCAGCGCCGATCGTGTCGTGCAGGGTCGCCGTGGTGTGGCCGCGACCAAGGCGGAGACGGCGCCCGCCTATATTACGACACCC |
|  | OL14593 | KKT4 S422E | Double-stranded DNA Repair Template Primer 1 | GCACCACGTTGGACACGTCTCGTCTGCAGGGCAGCGCCGATCGTGTCGTGCAGGGTCGCCGTGGTGTGGCCGCGACCAAGGCGGAGACGGAGCCCGCCTATATTACGACACCC |
|  | OL14594 | KKT4 S422S | Double-stranded DNA Repair Template Primer 1 | GCACCACGTTGGACACGTCTCGTCTGCAGGGCAGCGCCGATCGTGTCGTGCAGGGTCGCCGTGGTGTGGCCGCGACCAAGGCGGAGACGAGCCCCGCCTATATTACGACACCC |
|  | OL14595 | KKT4 S422A, S422E and S422S | Shared Double-stranded DNA Repair Template Primer 2 | TGAGTGCGCGTGCCGACGAGCGCGGTGGACGCCTTGCCGGCCGGCGTGGGTGTCGTAATATAGGCG |
|  | OL14612 | KKT4 S422 | WT Screening Primer 1 | GTGGTGATGTACGCCGGAGA |
|  | OL14613 | KKT4 S422A | Mutant Screening Primer 1 | GTCGTAATATAGGCGGGCGC |
|  | OL14614 | KKT4 S422E | Mutant Screening Primer 1 | GTCGTAATATAGGCGGGCTC |
|  | OL14615 | KKT4 S422S | Mutant Screening Primer 1 | GTCGTAATATAGGCGGGGCT |
|  | OL12870 | KKT4 S422 | Shared primer 2 | AGGGGGACCTTGTTGACGAT |
|  |  |  |  |  |
|  | OL14600 | KKT7 S304 | sgRNA | gaaattaatacgactcactataggGCTCTTCGCGAGGGCGTCGGgttttagagctagaaatagc |
|  | OL14596 | KKT7 S304A | Double-stranded DNA Repair Template Primer 1 | TCTAGACGCGCGCGCTTGCGGGTGTCGTTGTCGTCACCGTCTTCGTCCTCGCCCGATTTGGCCAGTGCATCCGCTGCGACAATGGAGATGGAGCGTC |
|  | OL14597 | KKT7 S304E | Double-stranded DNA Repair Template Primer 1 | TCTAGACGCGCGCGCTTGCGGGTGTCGTTGTCGTCACCGTCTTCGTCCTCGCCCGATTTGGCCAGTGCATCCGCTTCGACAATGGAGATGGAGCGTC |
|  | OL14598 | KKT7 S304S | Double-stranded DNA Repair Template Primer 1 | TCTAGACGCGCGCGCTTGCGGGTGTCGTTGTCGTCACCGTCTTCGTCCTCGCCCGATTTGGCCAGTGCATCCGCTGAGACAATGGAGATGGAGCGTC |
|  | OL14599 | KKT7 S304A, S304E and S304S | Shared Double-stranded DNA Repair Template Primer 2 | AGCTCGCACGCAACTCCATGGCGAAGCAACAGAGTCGCGTTCACTCTTCTGCTCACCAACGCCGACGCTCCATCTCCATTGTC |
|  | OL14617 | KKT7 S304 | WT Screening Primer 1 | TCGGCGGAGACAATGGATA |
|  | OL14618 | KKT7 S304A | Mutant Screening Primer 1 | GCCAGTGCATCCGCTGC |
|  | OL14619 | KKT7 S304E | Mutant Screening Primer 1 | GGCCAGTGCATCCGCTTC |
|  | OL14620 | KKT7 S304S | Mutant Screening Primer 1 | GCCAGTGCATCCGCTGA |
|  | OL14616 | KKT7 S304 | Shared primer 2 | CCAATAGTCGTCAGCGCCTT |
|  | OL12907 | KKT7 S304A, S304E and S304S | Sequencing PCR Forward | CATTCTGCCTGTGAACCAGC |
|  | OL12908 | KKT7 S304A, S304E and S304S | Sequencing PCR Reverse | GCTCTTGCTCTTGGCCTTCT |
|  |  |  |  |  |
| Used in Both | OL6137 / G00 | - | Universal Guide Primer | aaaagcaccgactcggtgccactttttcaagttgataacggactagccttattttaacttgctatttctagctctaaaac |
|  | OL12987 | KKT2 S493 | sgRNA | gaaattaatacgactcactataggGCACTGATCGAGACGTGCGAgttttagagctagaaatagc |
|  | OL12988 | KKT2 S493 | sgRNA | gaaattaatacgactcactataggAGCTTAACGGAGCAGGAGCGgttttagagctagaaatagc |
|  | OL12778 | KKT2 S923 | sgRNA | gaaattaatacgactcactataggGGTGTGCCCGCTGACGGAGGgttttagagctagaaatagc |
|  | OL12899 | KKT2 S923 | sgRNA | gaaattaatacgactcactataggAGCGGGCACACCGCACAGGGgttttagagctagaaatagc |
|  | OL12764 | KKT4 S422 | sgRNA | gaaattaatacgactcactataggATCGTGTCGTGCAAGGACGGgttttagagctagaaatagc |
|  | OL12900 | KKT2 S422 | sgRNA | gaaattaatacgactcactataggGTACATCACCACCCCCACGCgttttagagctagaaatagc |
|  | OL12825 | KKT7 S304 | sgRNA | gaaattaatacgactcactataggGCTCTTCGCGAGGGCATCCGCCGgttttagagctagaaatagc |
|  |  |  |  |  |
|  | OL12868 | KKT2 S923 | Forward Screening Primer – PCR construct for restriction digest | CGGAAGTCATCACGATCCGC |
|  |  |  | Sequencing Primer 1 |  |
|  | OL12869 | KKT2 S923 | Reverse Screening Primer – PCR construct for restriction digest | TTCTTGGGAGGAATCGCAGC |
|  |  |  | Sequencing Primer 2 |  |
|  |  |  |  |  |
|  | OL12906 | KKT4 S422A | Forward Screening Primer – PCR construct for restriction digest | ATTCACCGCAAAGACGAGGT |
|  |  | KKT4 S422A, S422E and S422S | Sequencing PCR Forward |  |
|  | OL12871 | KKT4 S422A | Reverse Screening Primer – PCR construct for restriction digest | TGTTGCGAGCGGATTCTGTT |
|  |  | KKT4 S422A, S422E and S422S | Sequencing PCR Reverse |  |

# Supplementary Table 3

Alignment of all repair template designs for each target site. Black text indicates protein sequence and WT DNA sequence. Orange text indicates synonymous mutations. Yellow highlight indicates target site. Blue text indicates nonsynonymous mutation. “–“ indicates an incomplete codon so translation has not been shown.

| KKT2 S25 WT Translation | - | P | L | M | S | H | F | C | G | S | L | S | R | T | P | P | R | G | G | A | I | S | M | P | R | D | L | S/A/E | Q | T | P | A | I | S | R | L | G | S | T | V | K | T | P | H | I | Q | K | C | V | V | D | Q | A | - |
| --- | --- | --- | --- | --- | --- | --- | --- | --- | --- | --- | --- | --- | --- | --- | --- | --- | --- | --- | --- | --- | --- | --- | --- | --- | --- | --- | --- | --- | --- | --- | --- | --- | --- | --- | --- | --- | --- | --- | --- | --- | --- | --- | --- | --- | --- | --- | --- | --- | --- | --- | --- | --- | --- | --- |
| KKT2 S25 WT sequence | GG | CCT | CTG | ATG | TCA | CAC | TTT | TGC | GGC | TCG | TTG | TCG | AGG | ACT | CCA | CCA | CGG | GGT | GGG | GCG | ATA | TCT | ATG | CCG | CGT | GAT | TTG | TCG | CAG | ACC | CCC | GCC | ATC | TCT | CGA | CTT | GGA | AGC | ACG | GTG | AAG | ACG | CCG | CAC | ATC | CAA | AAA | TGT | GTT | GTT | GAC | CAA | GCA | GA |
| KKT2 S25A dsDNA repair | GG | CCT | CTG | ATG | TCA | CAC | TTT | TGC | GGC | TCG | TTG | TCG | AGG | ACT | CCA | CCA | CGG | GGT | GGG | GCG | ATA | TCT | ATG | CCC | AGA | GAC | CTT | GCG | CAA | ACA | CCA | GCG | ATT | TCA | CGC | CTG | GGG | AGC | ACG | GTG | AAG | ACG | CCG | CAC | ATC | CAA | AAA | TGT | GTT | GTT | GAC | CAA | GCA | GA |
| KKT2 S25E dsDNA repair | GG | CCT | CTG | ATG | TCA | CAC | TTT | TGC | GGC | TCG | TTG | TCG | AGG | ACT | CCA | CCA | CGG | GGT | GGG | GCG | ATA | TCT | ATG | CCC | AGA | GAC | CTT | GAG | CAA | ACA | CCA | GCG | ATT | TCA | CGC | CTG | GGG | AGC | ACG | GTG | AAG | ACG | CCG | CAC | ATC | CAA | AAA | TGT | GTT | GTT | GAC | CAA | GCA | GA |
| KKT2 S25S dsDNA repair | GG | CCT | CTG | ATG | TCA | CAC | TTT | TGC | GGC | TCG | TTG | TCG | AGG | ACT | CCA | CCA | CGG | GGT | GGG | GCG | ATA | TCT | ATG | CCC | AGA | GAC | CTT | AGT | CAA | ACA | CCA | GCG | ATT | TCA | CGC | CTG | GGG | AGC | ACG | GTG | AAG | ACG | CCG | CAC | ATC | CAA | AAA | TGT | GTT | GTT | GAC | CAA | GCA | GA |
|  |  |  |  |  |  |  |  |  |  |  |  |  |  |  |  |  |  |  |  |  |  |  |  |  |  |  |  |  |  |  |  |  |  |  |  |  |  |  |  |  |  |  |  |  |  |  |  |  |  |  |  |  |  |  |
|  | - | C | G | S | V | S | L | V | S | E | V | A | D | R | E | E | A | A | P | R | T | S | R | S | V | R | R | S | V | S/A/E | L | T | E | Q | E | R | G | R | L | V | R | S | S | P | V | Q | Y | A | V | V | Y | P | G | - |
| KKT2 S493 WT sequence | GG | TGT | GGC | AGT | GTC | TCA | CTG | GTC | TCA | GAG | GTT | GCA | GAT | CGC | GAG | GAA | GCC | GCC | CCT | CGC | ACG | TCT | CGA | TCA | GTG | CGT | CGT | AGC | GTC | AGC | TTA | ACG | GAG | CAG | GAG | CGG | GGC | AGA | CTT | GTG | CGT | TCT | AGC | CCG | GTC | CAG | TAC | GCA | GTG | GTG | TAC | CCG | GGG | CG |
| KKT2 S493A ssODN |  |  |  |  |  |  |  | GTC | TCA | GAG | GTT | GCA | GAT | CGC | GAG | GAA | GCC | GCG | CCA | CGG | ACC | TCA | CGT | TCT | GTC | CGT | CGT | AGC | GTA | GCG | CTA | ACC | GAA | CAA | GAA | CGT | GGC | AGA | CTT | GTG | CGT | TCT | AGC | CCG | GTC | CAG | TAC |  |  |  |  |  |  |  |
| KKT2 S493A dsDNA repair | GG | TGT | GGC | AGT | GTC | TCA | CTG | GTC | TCA | GAG | GTT | GCA | GAT | CGC | GAG | GAA | GCC | GCG | CCA | CGG | ACC | TCA | CGT | TCT | GTC | CGT | CGT | AGC | GTA | GCG | CTA | ACC | GAA | CAA | GAA | CGT | GGC | AGA | CTT | GTG | CGT | TCT | AGC | CCG | GTC | CAG | TAC | GCA | GTG | GTG | TAC | CCG | GGG | CG |
| KKT2 S493E dsDNA repair | GG | TGT | GGC | AGT | GTC | TCA | CTG | GTC | TCA | GAG | GTT | GCA | GAT | CGC | GAG | GAA | GCC | GCG | CCA | CGG | ACC | TCA | CGT | TCT | GTC | CGT | CGT | AGC | GTA | GAG | CTA | ACC | GAA | CAA | GAA | CGT | GGC | AGA | CTT | GTG | CGT | TCT | AGC | CCG | GTC | CAG | TAC | GCA | GTG | GTG | TAC | CCG | GGG | CG |
| KKT2 S493S dsDNA repair | GG | TGT | GGC | AGT | GTC | TCA | CTG | GTC | TCA | GAG | GTT | GCA | GAT | CGC | GAG | GAA | GCC | GCG | CCA | CGG | ACC | TCA | CGT | TCT | GTC | CGT | CGT | AGC | GTA | TCT | CTA | ACC | GAA | CAA | GAA | CGT | GGC | AGA | CTT | GTG | CGT | TCT | AGC | CCG | GTC | CAG | TAC | GCA | GTG | GTG | TAC | CCG | GGG | CG |
|  |  |  |  |  |  |  |  |  |  |  |  |  |  |  |  |  |  |  |  |  |  |  |  |  |  |  |  |  |  |  |  |  |  |  |  |  |  |  |  |  |  |  |  |  |  |  |  |  |  |  |  |  |  |  |
| KKT2 S530 WT Translation |  | R | S | S | P | V | Q | Y | A | V | V | Y | P | G | R | D | T | A | T | R | W | N | L | R | A | V | V | S/A/E | L | P | R | D | M | T | D | E | I | E | R | E | F | K | C | M | N | G | H | V | M | T | K | L | T | S |
| KKT2 S530 WT sequence |  | CGT | TCT | AGC | CCG | GTC | CAG | TAC | GCA | GTG | GTG | TAC | CCG | GGG | CGC | GAC | ACT | GCC | ACT | CGT | TGG | AAC | CTT | CGC | GCC | GTA | GTA | TCG | CTG | CCA | CGC | GAC | ATG | ACG | GAC | GAG | ATC | GAG | CGC | GAG | TTC | AAG | TGC | ATG | AAC | GGG | CAC | GTA | ATG | ACA | AAG | TTG | ACC | TCG |
| KKT2 S530A dsDNA repair |  | CGT | TCT | AGC | CCG | GTC | CAG | TAC | GCA | GTG | GTG | TAC | CCG | GGG | CGC | GAC | ACT | GCG | ACA | CGG | TGG | AAT | TTG | CGG | GCG | GTT | GTA | GCG | CTC | CCT | CGG | GAT | ATG | ACC | GAT | GAA | ATT | GAA | CGC | GAG | TTC | AAG | TGC | ATG | AAC | GGG | CAC | GTA | ATG | ACA | AAG | TTG | ACC | TCG |
| KKT2 S530E dsDNA repair |  | CGT | TCT | AGC | CCG | GTC | CAG | TAC | GCA | GTG | GTG | TAC | CCG | GGG | CGC | GAC | ACT | GCG | ACA | CGG | TGG | AAT | TTG | CGG | GCG | GTT | GTA | GAG | CTC | CCT | CGG | GAT | ATG | ACC | GAT | GAA | ATT | GAA | CGC | GAG | TTC | AAG | TGC | ATG | AAC | GGG | CAC | GTA | ATG | ACA | AAG | TTG | ACC | TCG |
| KKT2 S530S dsDNA repair |  | CGT | TCT | AGC | CCG | GTC | CAG | TAC | GCA | GTG | GTG | TAC | CCG | GGG | CGC | GAC | ACT | GCG | ACA | CGG | TGG | AAT | TTG | CGG | GCG | GTT | GTA | AGT | CTC | CCT | CGG | GAT | ATG | ACC | GAT | GAA | ATT | GAA | CGC | GAG | TTC | AAG | TGC | ATG | AAC | GGG | CAC | GTA | ATG | ACA | AAG | TTG | ACC | TCG |
|  |  |  |  |  |  |  |  |  |  |  |  |  |  |  |  |  |  |  |  |  |  |  |  |  |  |  |  |  |  |  |  |  |  |  |  |  |  |  |  |  |  |  |  |  |  |  |  |  |  |  |  |  |  |  |
| KKT2 S923 WT Translation | - | V | E | E | H | V | V | K | Q | A | I | M | P | P | Q | V | P | R | G | R | A | Q | Q | P | R | A | P | S/A/E | V | S | G | H | T | A | Q | G | G | P | P | L | P | R | R | G | P | A | A | P | S | P | A | A | A | - |
| KKT2 S923 WT sequence | CT | GTG | GAG | GAG | CAC | GTG | GTG | AAG | CAA | GCC | ATC | ATG | CCG | CCT | CAG | GTG | CCA | CGC | GGA | CGA | GCA | CAG | CAG | CCA | CGT | GCC | CCC | TCC | GTC | AGC | GGG | CAC | ACC | GCA | CAG | GGC | GGT | CCG | CCA | CTG | CCG | CGC | CGC | GGC | CCA | GCT | GCG | CCA | TCT | CCT | GCA | GCC | GCT | TT |
| KKT2 S923A ssODN |  |  |  |  |  |  |  | AAG | CAA | GCC | ATC | ATG | CCG | CCT | CAA | GTG | CCA | CGC | GGA | CGA | GCA | CAG | CAG | CCA | CGT | GCG | CCA | GCG | GTT | TCG | GGT | CAT | ACG | GCT | CAA | GGT | GGT | CCG | CCA | CTG | CCG | CGC | CGC | GGC | CCA | GCT | GCG |  |  |  |  |  |  |  |
| KKT2 S923A dsDNA repair | CT | GTG | GAG | GAG | CAC | GTG | GTG | AAG | CAA | GCC | ATC | ATG | CCG | CCT | CAA | GTG | CCA | CGC | GGA | CGA | GCA | CAG | CAG | CCA | CGT | GCG | CCA | GCG | GTT | TCG | GGT | CAT | ACG | GCT | CAA | GGT | GGT | CCG | CCA | CTG | CCG | CGC | CGC | GGC | CCA | GCT | GCG | CCA | TCT | CCT | GCA | GCC | GCT | TT |
| KKT2 S923E dsDNA repair | CT | GTG | GAG | GAG | CAC | GTG | GTG | AAG | CAA | GCC | ATC | ATG | CCG | CCT | CAA | GTG | CCA | CGC | GGA | CGA | GCA | CAG | CAG | CCA | CGT | GCG | CCA | GAG | GTT | TCG | GGT | CAT | ACG | GCT | CAA | GGT | GGT | CCG | CCA | CTG | CCG | CGC | CGC | GGC | CCA | GCT | GCG | CCA | TCT | CCT | GCA | GCC | GCT | TT |
| KKT2 S923S dsDNA repair | CT | GTG | GAG | GAG | CAC | GTG | GTG | AAG | CAA | GCC | ATC | ATG | CCG | CCT | CAA | GTG | CCA | CGC | GGA | CGA | GCA | CAG | CAG | CCA | CGT | GCG | CCA | AGT | GTT | TCG | GGT | CAT | ACG | GCT | CAA | GGT | GGT | CCG | CCA | CTG | CCG | CGC | CGC | GGC | CCA | GCT | GCG | CCA | TCT | CCT | GCA | GCC | GCT | TT |
|  |  |  |  |  |  |  |  |  |  |  |  |  |  |  |  |  |  |  |  |  |  |  |  |  |  |  |  |  |  |  |  |  |  |  |  |  |  |  |  |  |  |  |  |  |  |  |  |  |  |  |  |  |  |  |
| KKT4 S422 WT Translation | - | T | T | L | D | T | S | R | L | Q | G | S | A | D | R | V | V | Q | G | R | R | G | V | A | A | T | K | A | E | T | S/A/E | P | A | Y | I | T | T | P | T | P | A | G | K | A | S | T | A | L | V | G | T | R | T | - |
| KKT4 S422 WT Sequence | AC | ACC | ACG | TTG | GAC | ACG | TCT | CGT | CTG | CAG | GGC | AGC | GCC | GAT | CGT | GTC | GTG | CAA | GGA | CGG | AGG | GGC | GTT | GCG | GCG | ACC | AAG | GCG | GAG | ACG | TCT | CCG | GCG | TAC | ATC | ACC | ACC | CCC | ACG | CCG | GCC | GGC | AAG | GCG | TCC | ACC | GCG | CTC | GTC | GGC | ACG | CGC | ACT | CA |
| KKT4 S422A ssODN |  |  |  |  |  |  |  |  | CTG | CAG | GGC | AGC | GCC | GAT | CGT | GTC | GTC | CAG | GGG | CGT | CGT | GGC | GTT | GCG | GCG | ACC | AAG | GCG | GAG | ACG | GCG | CCG | GCC | TAT | ATT | ACG | ACG | CCC | ACG | CCC | GCC | GGC | AAG | GCG | TCC | ACC | GCG | CTC |  |  |  |  |  |  |
| KKT4 S422A dsDNA repair | AC | ACC | ACG | TTG | GAC | ACG | TCT | CGT | CTG | CAG | GGC | AGC | GCC | GAT | CGT | GTC | GTG | CAG | GGT | CGC | CGT | GGT | GTG | GCC | GCG | ACC | AAG | GCG | GAG | ACG | GCG | CCC | GCC | TAT | ATT | ACG | ACA | CCC | ACG | CCG | GCC | GGC | AAG | GCG | TCC | ACC | GCG | CTC | GTC | GGC | ACG | CGC | ACT | CA |
| KKT4 S422E dsDNA repair | AC | ACC | ACG | TTG | GAC | ACG | TCT | CGT | CTG | CAG | GGC | AGC | GCC | GAT | CGT | GTC | GTG | CAG | GGT | CGC | CGT | GGT | GTG | GCC | GCG | ACC | AAG | GCG | GAG | ACG | GAG | CCC | GCC | TAT | ATT | ACG | ACA | CCC | ACG | CCG | GCC | GGC | AAG | GCG | TCC | ACC | GCG | CTC | GTC | GGC | ACG | CGC | ACT | CA |
| KKT4 S422S dsDNA repair | AC | ACC | ACG | TTG | GAC | ACG | TCT | CGT | CTG | CAG | GGC | AGC | GCC | GAT | CGT | GTC | GTG | CAG | GGT | CGC | CGT | GGT | GTG | GCC | GCG | ACC | AAG | GCG | GAG | ACG | AGC | CCC | GCC | TAT | ATT | ACG | ACA | CCC | ACG | CCG | GCC | GGC | AAG | GCG | TCC | ACC | GCG | CTC | GTC | GGC | ACG | CGC | ACT | CA |
|  |  |  |  |  |  |  |  |  |  |  |  |  |  |  |  |  |  |  |  |  |  |  |  |  |  |  |  |  |  |  |  |  |  |  |  |  |  |  |  |  |  |  |  |  |  |  |  |  |  |  |  |  |  |  |
| KKT7 S304 WT Translation | - | L | A | R | N | S | M | A | K | Q | Q | S | R | V | H | S | S | A | H | Q | R | R | R | S | I | S | I | V | S/A/E | A | D | A | L | A | K | S | G | E | D | E | D | G | D | D | N | D | T | R | K | R | A | R | L | - |
| KKT7 S304 WT Sequence | CT | CTC | GCA | CGC | AAC | TCC | ATG | GCG | AAG | CAA | CAG | AGT | CGC | GTT | CAC | TCT | TCT | GCC | CAT | CAG | CGT | CGT | CGG | TCG | ATA | TCC | ATT | GTC | TCC | GCC | GAC | GCC | CTC | GCG | AAG | AGC | GGC | GAG | GAC | GAA | GAC | GGT | GAC | GAC | AAC | GAC | ACC | CGC | AAG | CGC | GCG | CGT | CTA | GA |
| KKT7 S304A ssODN |  |  |  |  |  |  |  | GCG | AAG | CAA | CAG | AGT | CGC | GTT | CAC | TCT | TCT | GCG | CAC | CAA | CGG | CGG | CGT | AGC | ATT | AGC | ATT | GTC | GCG | GCG | GAT | GCG | CTT | GCC | AAA | TCG | GGC | GAG | GAC | GAA | GAC | GGT | GAC | GAC | AAC | GAC | ACC |  |  |  |  |  |  |  |
| KKT7 S304S ssODN |  |  |  |  |  |  |  | GCG | AAG | CAA | CAG | AGT | CGC | GTT | CAC | TCT | TCT | GCG | CAC | CAA | CGG | CGG | CGT | AGC | ATT | AGC | ATT | GTC | TCG | GCG | GAT | GCG | CTT | GCC | AAA | TCG | GGC | GAG | GAC | GAA | GAC | GGT | GAC | GAC | AAC | GAC | ACC |  |  |  |  |  |  |  |
| KKT7 S304A dsDNA repair | CT | CTC | GCA | CGC | AAC | TCC | ATG | GCG | AAG | CAA | CAG | AGT | CGC | GTT | CAC | TCT | TCT | GCT | CAC | CAA | CGC | CGA | CGC | TCC | ATC | TCC | ATT | GTC | GCA | GCG | GAT | GCA | CTG | GCC | AAA | TCG | GGC | GAG | GAC | GAA | GAC | GGT | GAC | GAC | AAC | GAC | ACC | CGC | AAG | CGC | GCG | CGT | CTA | GA |
| KKT7 S304E dsDNA repair | CT | CTC | GCA | CGC | AAC | TCC | ATG | GCG | AAG | CAA | CAG | AGT | CGC | GTT | CAC | TCT | TCT | GCT | CAC | CAA | CGC | CGA | CGC | TCC | ATC | TCC | ATT | GTC | GAA | GCG | GAT | GCA | CTG | GCC | AAA | TCG | GGC | GAG | GAC | GAA | GAC | GGT | GAC | GAC | AAC | GAC | ACC | CGC | AAG | CGC | GCG | CGT | CTA | GA |
| KKT7 S304S dsDNA repair | CT | CTC | GCA | CGC | AAC | TCC | ATG | GCG | AAG | CAA | CAG | AGT | CGC | GTT | CAC | TCT | TCT | GCT | CAC | CAA | CGC | CGA | CGC | TCC | ATC | TCC | ATT | GTC | TCA | GCG | GAT | GCA | CTG | GCC | AAA | TCG | GGC | GAG | GAC | GAA | GAC | GGT | GAC | GAC | AAC | GAC | ACC | CGC | AAG | CGC | GCG | CGT | CTA | GA |

# Supplementary Table 4

Key Primer3 settings used in the Python script. Other input settings required were the suggested settings based on the Primer3 documentation.

|  | Argument | Input for Repair Primers | Input for Screening Primers |
| --- | --- | --- | --- |
| Seq_args | SEQUENCE_TEMPLATE | Artificial integrated repair template sequence | Either WT input sequence (for WT primers) or artificial integrated repair template sequence (for mutant primers) |
|  | PRIMER_PICK_LEFT_PRIMER  PRIMER_PICK_RIGHT_PRIMER | 3 | 1 |
|  | PRIMER_OPT_SIZE | 18 | 18 |
|  | PRIMER_MIN_SIZE | 15 | 15 |
|  | PRIMER_MAX_SIZE | 24 | 22 |
|  | PRIMER_PRODICT_SIZE_RANGE | 100, 1000 | 150, 1500 |
|  | SEQUENCE_PRIMER_PAIR_OK_REGION_LIST | Forward – entire length of the recoded region of the repair template. Reverse – everything downstream of the repair template | Forward - Entire sequence before the start of the repair template. Reverse – start of the repair template to the end of the repair template |
|  | SEQUENCE_PRIMER | none | WT = none, mutant = WT forward primer |
| Global_args | PRIMER_MIN_SIZE | 16 | 18 |
|  | PRIMER_OPT_SIZE | 20 | 20 |
|  | PRIMER_MAX_SIZE | 27 | 27 |
|  | PRIMER_PRODUCT_OPT_SIZE | 0 | 500 |
|  | PRIMER_MIN_GC | 20.0 | 20.0 |
|  | PRIMER_OPT_GC_PERCENT | 50.0 | 50.0 |
|  | PRIMER_MAX_GC | 80.0 | 80.0 |
|  | PRIMER_MIN_TM | 55.0 | 56.0 |
|  | PRIMER_OPT_TM | 60.0 | 60.0 |
|  | PRIMER_MAX_TM | 67.0 | 63.0 |
